# Control of Mitochondrial Electron Transport Chain Flux and Apoptosis by Retinoic Acid: Raman Imaging In Vitro Human Bronchial and Lung Cancerous Cells

**DOI:** 10.3390/cancers15184535

**Published:** 2023-09-13

**Authors:** Halina Abramczyk, Jakub Maciej Surmacki

**Affiliations:** Laboratory of Laser Molecular Spectroscopy, Department of Chemistry, Institute of Applied Radiation Chemistry, Lodz University of Technology, Wroblewskiego 15, 93-590 Lodz, Poland

**Keywords:** Raman imaging, retinoic acid, electron transport chain, human bronchial (BEpC) and lung cancer (A549) cell line, apoptosis, cell signaling, cytochrome *c*, phosphorylation, reactive oxygen species, respiration

## Abstract

**Simple Summary:**

We have shown that a confocal Raman imaging provides an excellent tool to extend our knowledge on the redox status of iron ion of heme proteins inside specific organelles of bronchial normal epithelial lung (BEpC) and lung cancer (A549) cells. The role of retinoic acid in regulating respiration, apoptosis processes, and reactive oxygen species production has been discussed. We showed that retinoic acid plays a key role in the oscillations between reduced (Fe^2+^) and oxidized (Fe^3+^) form of cytochrome *c* in the electron transport chain that can activate or inhibit these physiological functions. The redox status of cytochrome *c* regulated by retinoic acid becomes a target for further development of robust therapeutic approaches.

**Abstract:**

The multiple functions of cytochrome *c* (cyt *c*) and their regulation in life and death decisions of the mammalian cell go beyond respiration, apoptosis, ROS scavenging, and oxidation of cardiolipine. It has become increasingly evident that cyt *c* is involved in the propagation of mitogenic signals. It has been proposed that the mitogenic signals occur via the PKCδ-retinoic acid signal complex comprising the protein kinase Cδ, the adapter protein Src homologous collagen homolog (p66Shc), and cyt *c*. We showed the importance of retinoic acid in regulating cellular processes monitored by the Raman bands of cyt *c*. To understand the role of retinoids in regulating redox status of cyt *c*, we recorded the Raman spectra and images of cells receiving redox stimuli by retinoic acid at in vitro cell cultures. For these purposes, we incubated bronchial normal epithelial lung (BEpC) and lung cancer cells (A549) with retinoic acid at concentrations of 1, 10, and 50 µM for 24 and 48 h of incubations. The new role of retinoic acid in a change of the redox status of iron ion in the heme group of cyt *c* from oxidized Fe^3+^ to reduced Fe^2+^ form may have serious consequences on ATPase effectiveness and aborting the activation of the conventional mitochondrial signaling protein-dependent pathways, lack of triggering programmed cell death through apoptosis, and lack of cytokine induction. To explain the effect of retinoids on the redox status of cyt *c* in the electron transfer chain, we used the quantum chemistry models of retinoid biology. It has been proposed that retinol catalyzes resonance energy transfer (RET) reactions in cyt *c*. The paper suggests that RET is pivotally important for mitochondrial energy homeostasis by controlling oxidative phosphorylation by switching between activation and inactivation of glycolysis and regulation of electron flux in the electron transport chain. The key role in this process is played by protein kinase C δ (PKCδ), which triggers a signal to the pyruvate dehydrogenase complex. The PKCδ-retinoic acid complex reversibly (at normal physiological conditions) or irreversibly (cancer) responds to the redox potential of cyt *c* that changes with the electron transfer chain flux.

## 1. Introduction

Glycolysis, Kreb’s Cycle, and the electron transport chain are the most important processes responsible for the life and death of living cells. Glycolysis is the first step in cellular homeostasis. Glycolysis occurs in the cytoplasm of the cell and a series of reactions produce pyruvic acids. The mitochondrial matrix enzyme pyruvate dehydrogenase complex (*PDHc*) links glycolysis to the Krebs cycle by converting pyruvate into acetyl-coenzyme A in the process of oxidative decarboxylation. Acetyl-CoA begins the Krebs cycle in mitochondria, by reacting with oxaloacetic acid in the presence of enzymes and creating the first intermediate—citric acid of the Krebs cycle. The citric acid is then oxidized over a number of stages in the Krebs cycle, which produces NADHs (nicotinamide adenine dinucleotide), FADH_2_ (flavin adenine dinucleotide), and ATP (adenosine triphosphate). NADH and FADH_2_ molecules provide electrons for the electron transport chain (ETC) and produce further ATPs. Electrons in ETC transfer energy to a series of protein complexes and other molecules in the membrane of mitochondria via redox reactions. The flow of electrons through the electron transport chain from a lower to a higher redox potential creates an electrochemical proton gradient that drives the synthesis of ATP via coupling oxidative phosphorylation with ATP synthase. Aerobic respiration culminates in the termination of electron flow with molecular oxygen as the final electron acceptor, whereas in anaerobic respiration, alternative electron acceptors, such as sulfate, are employed. This provides the energy that is required for the pumping of hydrogen ions across a membrane. The electron transport chain produces the majority of the ATPs during respiration. Cytochrome *c* (cyt *c*) is a key protein in the electron transport chain that is needed to maintain life through respiration, but also determines cell death through apoptosis. Cyt *c* is primarily known for its function in the mitochondria as a key participant in the mitochondrial electron transport that shuttles electrons between respiratory complexes III and IV.

Cyt *c* serves a vital role in maintaining a balance of cellular homeostasis, which is not limited to energy level maintenance but includes such diverse functions as oxidizing of cardiolipin during apoptosis, scavenging, and producing radicals. The balance between two seemingly contradictory functions determines life-sustaining respiration and apoptosis. Cyt *c* serves the dual function of scavenging reactive oxygen species (ROS) in normal conditions and generating ROS alongside the co-factor p66Shc [1]. When the detrimental effects of stress signals outweigh the advantages of sustaining cell viability, multiple signals can trigger the commencement of programmed cell death through intrinsic (mitochondrial) type II apoptosis.

There is growing evidence that phosphorylation of cyt *c* regulates mitochondrial respiration and apoptosis. So far, five phosphorylation sites of cyt *c* have been mapped and functionally characterized: *Tyr97*, *Tyr48*, *Thr28*, *Ser47*, and *Thr58* [2]. It was suggested that cyt *c* phosphorylation partially inhibits mitochondrial respiration [1]. Tyrosine phosphorylation is very specific and mainly found in higher organisms. It is this regulatory mechanism that affects cyt *c* in a distinct tissue-specific manner. In the past, commonly used isolation methods of cyt *c* have not taken into account the possibility of cyt *c* phosphorylation in vivo. Tyr48-phosphorylated cyt *c* functions as a more efficient peroxidase-like enzyme, especially when it is bound to liposomes containing cardiolipin [3].

It has become increasingly evident that cyt *c* is involved in the propagation of mitogenic signals. Within the PKCδ signal complex, one might find the protein kinase Cδ, the adapter protein Src homologous collagen homolog (p66Shc), and cyt *c* as its key constituents [4,5].

Cell signaling targeting cyt *c* and other mitochondrial proteins involved in oxidative phosphorylation is a new research area. It may transform the traditional thinking about the regulation of glycolysis, Krebs cycle, electron transport chain, and ATP synthesis, which are mainly derived from separated pathways. In this paper, we present the experimental results that suggest that there is one central mechanism integrating and regulating the diverse functions of cyt *c*. There is growing evidence that retinoic acid is a key player in coupling the triangle glycolysis-Krebs cycle-electron transport chain. Recently, we showed that retinoic acid and, to a lesser extent, retinol is pivotally important for mitochondrial energy homeostasis by controlling the redox status of cyt *c* in the electron transport chain controlling oxidative phosphorylation and apoptosis. We demonstrated that label-free Raman microscopy helps to clarify the precise role of retinoids in the metabolism and signaling of cancer cells. Our research aimed to elucidate the involvement of retinoids in various human cell lines, including normal astrocytes (NHA), high-grade glioblastoma tumor cells (U-87 MG), as well as tissues derived from individuals with medulloblastoma and glioblastoma [6]. We found that reduced cyt *c* accumulates in cancers, whereas oxidized cyt *c* prevails in normal cells.

At normal physiological conditions, cyt *c* is located in the intermembrane space of the mitochondrion. During pathological conditions, cyt *c* is released into the cytosol and triggers massive programmed cell death through apoptosis [7]. Cell death is an essential process for tissue development and renewal; however, excessive loss of cells can be a sign of disease.

We showed that cyt *c* plays a crucial role in the development and progression of cancer [8,9,10,11]. Cyt *c* in mitochondrion changes its redox status between oxidized and reduced forms, but at the stationary Raman measurements at the normal physiological conditions, both in tissue and in vitro cell cultures the oxidized form dominates. This balance changes and is shifted towards reduced form in cancers. Cyt *c* operates at a low, basal level in normal cells, but it is strongly induced to very high levels in pathological cancer states [8]. We found that the concentration of reduced cyt *c* becomes abnormally high in human brain tumors and breast cancers in human tissues. The second important finding is the striking trend for in vitro cells, which is opposite to that observed in the tissues. The concentration of cyt *c* becomes lower in cancer cells when compared to the normal cells at in vitro conditions. These findings offer convincing evidence of the tumor environment’s central role in cancer development. Over the last decade, this conclusion has been firmly established, emphasizing that cancer cells must be situated within the tumor microenvironment. This insight signifies that understanding tumor biology requires a more comprehensive approach, extending beyond the isolated study of cells in in vitro cultures. The results of our investigation confirm the importance of the tumor microenvironment, which incorporates essential components of cancer progression close to tumor cells, such as fibroblasts, immune cells, and the extracellular matrix. Furthermore, these findings suggest the existence of intracellular reductants in tissue beyond the reduced nicotinamide adenine dinucleotide (NADH) (present in the growing medium in cell culturing), contributing to the activity of cyt *c*.

Some hints on the essential reductants that enter into mitochondria from the microenvironment have been provided by detailed analysis of Raman spectra of various organelles in cancer cells by using different laser excitation wavelengths. This approach has generated Raman resonance enhancement for specific molecules that cannot be visible for non-resonance conditions. Our results demonstrated a significant Raman resonance enhancement at 355 nm where the family of retinoids have the absorption [6]. This finding was a reason we started to look carefully at retinoids as an essential factor in cancer development [6]. Retinoids appear to play an important role also in other important physiological processes such as immunity. It has been reported that retinoic acid is a key player in immunity. RIG-I (retinoic acid-inducible gene I) is a cytosolic pattern recognition receptor (PRR) responsible for the type-1 interferon (IFN1) response. RIG-I is an essential molecule in the innate immune system for recognizing cells that have been infected with a virus [12].

To understand the role of retinoids in cellular processes, we must use proper tools for sensing retinoids in vivo to monitor retinoid distribution in specific organelles. Raman imaging is an excellent tool that can not only provide a biochemical profile of the organelles in the cells but also control the redox status of cyt *c* in mitochondria and cytoplasm.

The study aimed to investigate the role of retinoids and cyt *c* in regulating glycolysis, Krebs cycle, and electron transport chain cellular signal transduction in cancers. To answer the question on the role of retinoids in the process of redox changes in cyt *c* in cancers, we recorded the Raman images of cells receiving redox stimuli by retinoic acid (RA) at in vitro cell cultures. For these purposes, we incubated human bronchial (BEpC) and lung cancer cells (A549 adenocarcinoma human alveolar basal epithelial cells) with retinoic acid (RA) at concentrations of RA 1, 10, and 50 µM) for 24 and 48 h of incubations.

## 2. Materials and Methods

### 2.1. Reference Chemicals

Cytochrome *c* (no. C2506) and retinoic acid (no. R2625) were purchased from Merck Life Science.

### 2.2. Cell Culture and Preparation for Raman Spectroscopy and Imaging

An A549 human lung carcinoma cell line (no. CCL-185, ATCC) and human bronchial epithelial cells (BEpC) (no. P10557, Innoprot) were used. A549 cells were grown in F-12K Medium (no. 30-2004, ATCC) with 10% fetal bovine serum (FBS no. 30-2020, ATCC) and maintained at 37 °C in a humidified atmosphere containing 5% CO_2_. BEpC cells were grown in Bronchial Epithelial Cell Medium (no. P60151, Innoprot) and maintained at 37 °C in a humidified atmosphere containing 5% CO_2_ according the Innoprot’s protocol. Cells were seeded on a CaF_2_ window (Crystran Ltd., Poole, UK; CaF_2_ Raman grade optically polished window 25 mm diameter × 1 mm thick, no. CAFP25-1R, Poole, UK) in a 35 mm Petri dish at a density of 5 × 10^4^ cells per Petri dish the day before the examination. Before Raman examination, cells were supplemented with retinoic acid (1, 10, and 50 μM) for 24 and 48 h, then fixed with 4% formalin solution (neutrally buffered) and kept in PBS (no. 10010023, Gibco) during the experiment. For each culture condition, at least 3 cells were imaged with a minimum number of Raman spectra of 1600 for a single cell. After conducting Raman imaging measurements, the cells were treated with Hoechst 33342 (25 μL at 1 μg/mL per mL of PBS; images collection: excitation: 355 nm, integration time: 0.01 s, resolution: 1 μm) and Oil Red O (10 μL of 0.5 mM Oil Red dissolved in 60% isopropanol/dH_2_O per each mL of PBS; images collection: excitation: 532 nm, integration time: 0.01 s, resolution: 1 μm) through a 15 min incubation. Following a PBS wash, the cells were imaged for fluorescence using an Alpha 300RSA WITec microscope, with the addition of fresh PBS.

### 2.3. Raman Spectroscopy and Imaging

Raman measurements of the human breast adenocarcinoma were conducted on a WITec confocal alpha 300 Raman microscope with the use of a 532 nm excitation wavelength coupled to the microscope via an optical fiber (50 μm diameter). The 40× objective (NIKON CFI Plan Fluor C ELWD (Extra-Long Working Distance) 40×: N.A. 0.60, W.D. 3.6–2.8 mm; DIC-M, C.C.0-2) was used. A standard alignment procedure (single-point calibration) was performed before the collection of Raman spectra with the use of Raman scattering vibration produced by a silicon plate (520.7 cm^−1^). The spectra were measured with a 532 nm excitation wavelength laser with the power of 10 mW in the focus spot and with an integration time of 0.3 s by Andor Newton DU970-UVB-353 CCD camera in enhanced mode (EMCCD). Raman data analysis was performed using WITec (WITec Project Plus 4) and OriginPro 2018 programs. Raman imaging data were analyzed by the Cluster Analysis method described in [9,10,13]. Raman maps presented in the manuscript were constructed based on principles of Cluster Analysis described in detail in [9,10,13]. The number of clusters was 6 (the minimum number of clusters characterized by different average Raman spectra, which describe the organelles in the cell: nucleus, lipid droplets/ER, cytoplasm, mitochondria, cell border). The colors of the clusters correspond to the colors of the Raman spectra of nucleus (red), lipid droplets (orange), endoplasmic reticulum (blue), cytoplasm (green), mitochondria (magenta), and cell border (light grey).

Number of analyzed cells *n*(BEpC) = 6, *n*(BEpC with retinoic acid) = 19, *n*(A549) = 6, *n*(A549 with retinoic acid) = 18; number of control and incubated with retinoic acid Raman spectra of BEpC and A549 used for averaging 10,850, 28,525, and 18,225, 38,500, respectively. 

### 2.4. ANOVA

The statistical analysis of the spectroscopic data was performed by using the one-way ANOVA test implemented in OriginPro 2016 software. The Tukey test was used to calculate the value of statistical significance; asterisk * denotes that the differences are statistically significant, *p*-value  ≤  0.05.

## 3. Results and Discussion

We will concentrate on the redox status of cyt *c* in the specific organelles inside human lung cancer cells in vitro. The redox status of cyt *c* in mitochondria plays a crucial role in maintaining a balance of cellular homeostasis. Cyt *c* is primarily known for its function in the mitochondria as a key participant in the mitochondrial electron transport that shuttles electrons between respiratory complexes III and IV. Cyt *bc_1_* from the complex III donates one electron to the oxidized form of cyt c (Fe^3+^), reducing the iron of cyt *c* by one oxidation state to become Fe^2+^. The reduced cyt c may then be rapidly reoxidized by cyt *c* oxidase (complex IV) to regenerate oxidized cyt *c* [8,9,10,11]. The oxidized cyt *c* is rapidly reduced in the cytosol where it can activate the apoptosome and initiate apoptosis [14].

Therefore, intracellular reductants in cells play an important role in mechanisms regulating the propagation of mitogenic and cytosolic signals. It has become increasingly evident that retinoic acid and retinol are involved in the propagation of mitogenic signals via alterations of cyt *c* redox status [5,6]. Moreover, it has been reported that retinoic acid is a key player in immunity RIG-I [12,15]. 

To study mitochondrial processes in cells, we applied Resonance Raman imaging to probe the redox status of cytochrome in mitochondria, cytoplasm, lipid droplets, and nucleus. The Resonance Raman spectroscopy is a remarkably effective probe for to study heme proteins. At non-resonant conditions, Raman signals of cyt *c* (present at approx. 1 mM in the IMS (intermembrane space of mitochondrion) or even less (nM)) [16,17,18] are hidden by stronger signals of other proteins, lipids, and DNA. However, at the laser excitations corresponding to the electron resonances of the Sorret or Q band transitions, the Raman signal increases a few orders and the Raman spectrum of cells and tissues is dominated by heme protein vibrations. Moreover, Resonance Raman spectroscopy allows us to distinguish between the oxidized and reduced cyt *c*.

**Figure 1:**
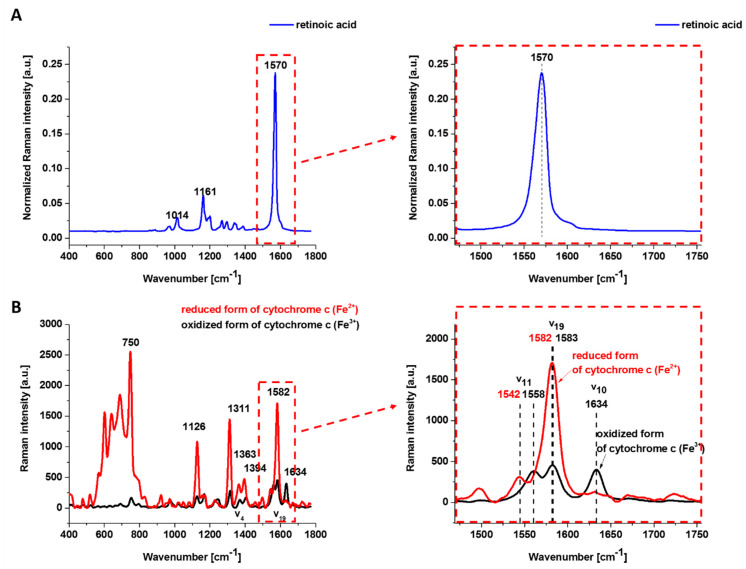
Raman spectra of retinoic acid (normalized) (**A**) and isolated cytochrome c (**B**). Isolated cytochrome c in solution (0.23 mM) dissolved potassium phosphate buffer (pH 7.3), ferric (Fe^3+^, oxidized (black)) and ferrous form (Fe^2+^, reduced (red)) of cytochrome c. Ferrous cytochrome c was prepared by adding a 10-fold excess of reduction agent—ascorbic acid. Excitation: 532 nm, laser power: 10 mW, integration time: 0.5 s, accumulation: 10.

Figure 1 shows the Raman spectrum of retinoic acid and the Resonance Raman spectrum of isolated oxidized (Fe^3+^) and reduced (Fe^2+^) form of cyt *c* excited with laser 532 nm being in resonance with the electron Q band. The studied concentrations of retinoic acid and laser excitation at 532 nm have a negligible Raman signal.

One can see from Figure 1B that the reduced cyt *c* (Fe^2+^) has a much higher intensity of the Raman bands than the oxidized cyt *c* (Fe^3+^). The Raman band *㯘*_19_ of cyt *c* corresponding to the methine bridge vibrations is only slightly sensitive to the oxidation—state of iron ion and appears at 1582 cm^−1^ for the reduced and at 1583 cm^−1^ for the oxidized cyt *c*. In contrast, the Raman band ν_11_ of cyt *c* is sensitive to the oxidation state of iron ion and appears at 1542 cm^−1^ for the reduced Fe^2+^ cyt *c* and 1558 cm^−1^ for the oxidized Fe^3+^ cyt *c*.

The 𝐵_1𝑔_ mode, i.e., 1634 cm^−1^ (*㯘*_10_), which is relatively strong in comparison with the other vibrations of the oxidized cyt *c,* disappears for the reduced cyt *c*. 

Having the method that will allow us to probe the redox status of cytochrome in living mitochondria, cytoplasm, lipid droplets, and endoplasmic reticulum, we will focus on human lung cancer cells. To improve our knowledge of the role of retinoids in the process of redox changes in cyt *c,* we recorded the Raman spectra of cells receiving redox stimuli by RA in the absence of cell-to-cell interactions at in vitro cell cultures. For these purposes, we incubated human lung cancer cells A549 (adenocarcinoma cell) and bronchial normal epithelial lung cells (BEpC) with RA at concentrations of 1, 10, and 50 μM for 24 and 48 h of incubations. 

**Figure 2:**
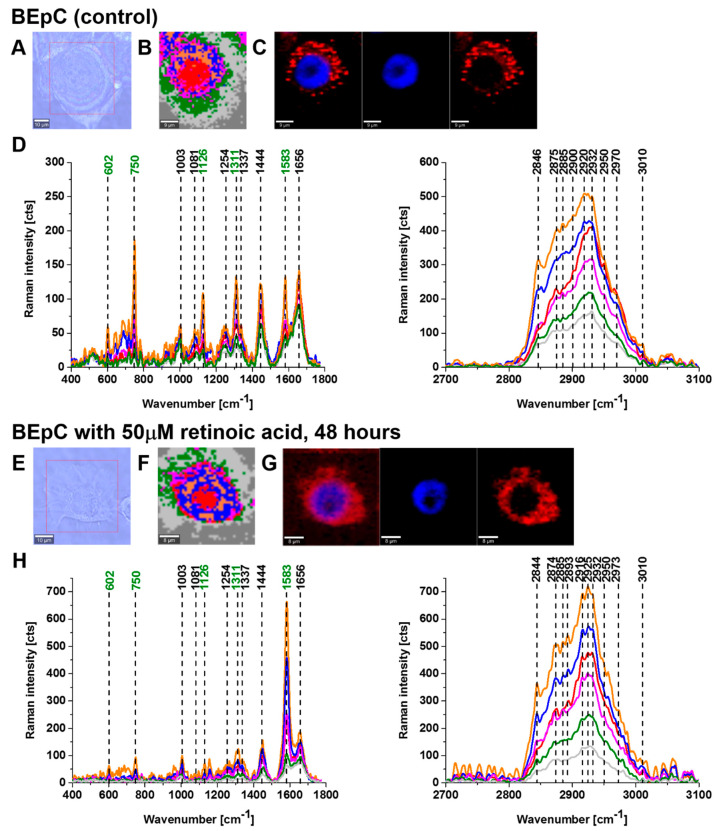
Raman imaging of a typical human bronchial epithelial lung cell (BEpC) without (control) and incubated with retinoic acid (50 μM) for 48 h. Microscopy images (**A**,**E**), Raman images with the respective Raman spectra (**B**,**D**,**F**,**H**) of a nucleus (red), lipid droplets (orange), endoplasmic reticulum (blue), cytoplasm (green), mitochondria (magenta), cell border (light grey). Fluorescence images (**C**,**G**). Resolution of Raman images 1 μm, integration time 0.3 s, 10 mW at 532 nm and resolution of fluorescence images 1 μm, integration time 0.01 s (Nucleus—blue (Hoechst 33342), ER/lipid droplets—red (Oil Red O)).

**Figure 3:**
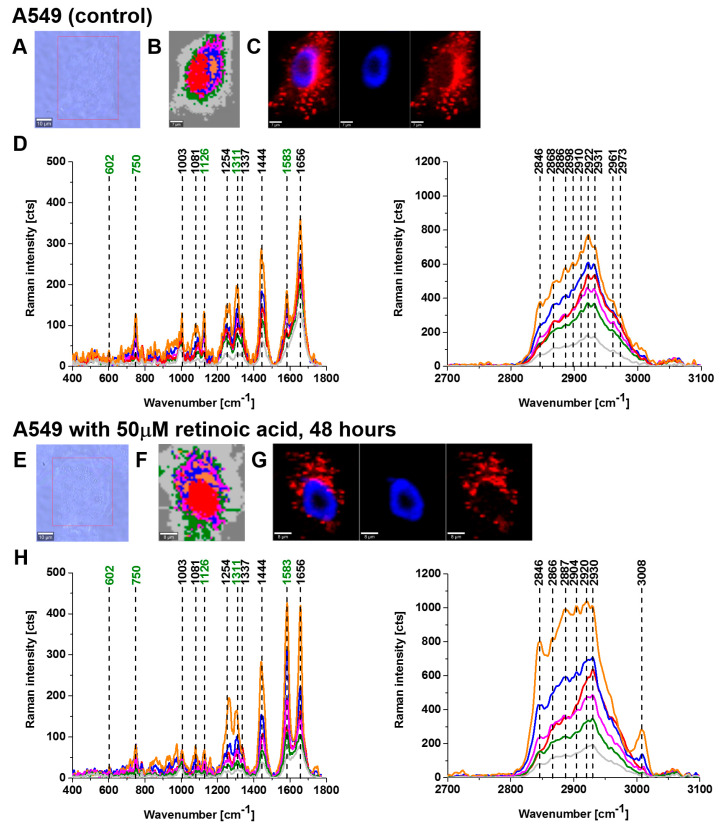
Raman imaging of a typical lung cancer cells (A549) without (control) and incubated with retinoic acid (50 μM) for 48 h. Microscopy images (**A**,**E**), Raman images with the respective Raman spectra (**B**,**D**,**F**,**H**) of a nucleus (red), lipid droplets (orange), endoplasmic reticulum (blue), cytoplasm (green), mitochondria (magenta), cell border (light grey). Fluorescence images (**C**,**G**). Resolution of Raman images 1 μm, integration time 0.3 s, 10 mW at 532 nm and resolution of fluorescence images 1 μm, integration time 0.01 s (Nucleus—blue (Hoechst 33342), ER/lipid droplets—red (Oil Red O)).

Figure 2 and Figure 3 show imaging analysis of bronchial (BEpC) and lung cancer cells (A549) by Raman and fluorescence spectroscopy.

Figure 2H and Figure 3H show the Raman spectra in typical BEpC and A549 cells incubated with retinoic acid (50 µM, incubated for 48 h). The bands at 750, 1126, 1311, and 1582 cm^−1^ correspond to the heme group of cyt *c*, the band at 1656 cm^−1^ represents the Amide I vibration of the cell. One can see that cyt *c* is localized in mitochondria, endoplasmic reticulum, lipid droplets, and cytoplasm. To determine the effect of retinoic acid on redox status of cyt *c* in specific organelles, we compared the Raman spectra of control cells (without retinoic acid) with the cells receiving redox stimuli by retinoic acid at in vitro cell cultures. 

**Figure 4:**
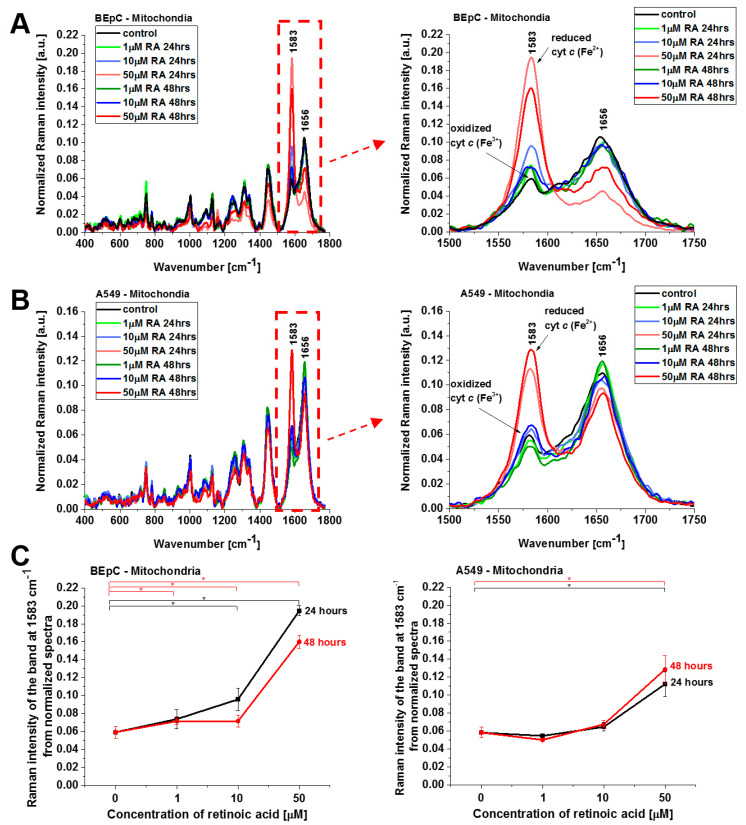
Normalized average Raman spectra of mitochondria of human bronchial (BEpC) (**A**) and lung cancer cells (A549) (**B**) cells without and incubated with retinoic acid (1, 10, 50 μM) for 24 and 48 h. (**C**) Raman band ν_19_ intensity at 1583 cm^−1^ as a function of retinoic acid concentration in mitochondria of the BEpC and A549 cells. The concentration *c* = 0 μM represents the control cells without retinoic acid.

Figure 4 shows normalized average Raman spectra (normalized by vector norm) in the range 400–1800 cm^−1^ for the mitochondria of a human bronchial (BEpC) and cancer lung cell (A549) without (control) and incubated with retinoic acid at a concentration of 1, 10, and 50 μM for 24 and 48 h. It is striking that a dramatic increase in the Raman signal of the ν_19_ band at 1583 cm^−1^ of cyt *c* is observed upon incubation with retinoic acid. This enhancement illustrates the change in the redox status of the heme group from the oxidized iron ion Fe^3+^ to the reduced Fe^2+^ as we showed in Figure 3B that the Raman bands of the reduced form of cyt *c* have much higher intensities than those of the oxidized form. Figure 4C shows Raman band ν_19_ intensity at 1583 cm^−1^ as a function of retinoic acid concentration. One can see that at low concentrations of retinoic acid, up to 10 µM, the intensity of the Raman signal in mitochondrion does not change significantly, indicating that the cyt *c* still remains in the oxidized state with the Fe^3+^ iron ion of the heme group. For higher concentrations, retinoic acid changes the redox status of cyt *c* spectacularly to the reduced Fe^2+^. The physiological concentrations of retinoic acid in the blood are around 3–140 nM [19]. Our results show that a concentration of 50 μM is likely to overwhelm normal metabolism and might elicit retinoid toxicity responses. Kéri et al. found that 10 µM of all-*trans* retinoic acid (ATRA) treatment decreased the proliferation rate and the tyrosine kinase activity of SW620, HT29, and COLO205, while SW480 cells showed resistance to ATRA treatment [20,21].

**Figure 5:**
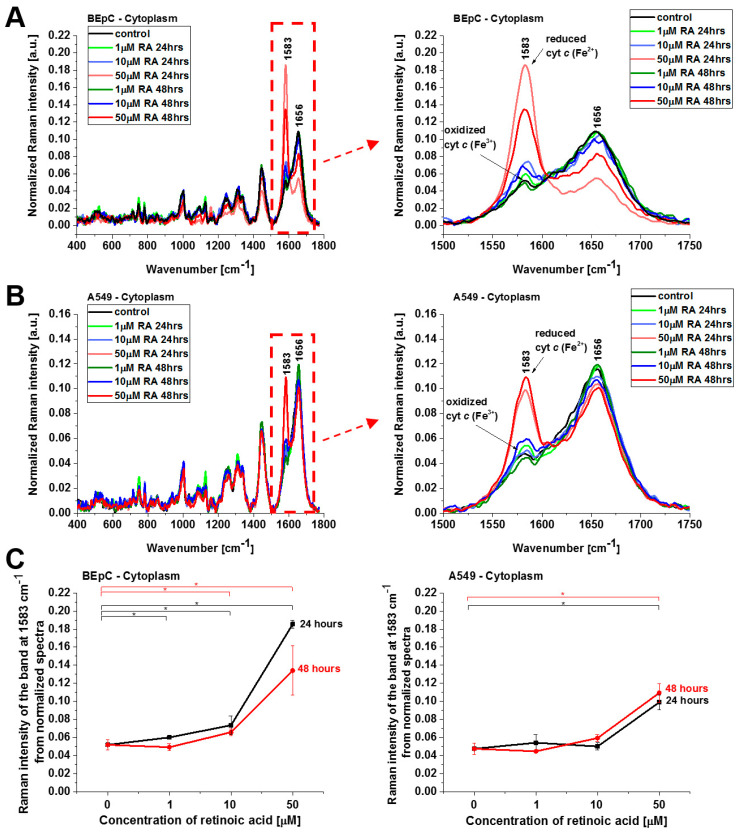
Normalized average Raman spectra of cytoplasm of human bronchial (BEpC) (**A**) and lung cancer cells (A549) (**B**) cells without and incubated with retinoic acid (1, 10, 50 μM) for 24 and 48 h. (**C**) Raman band ν_19_ intensity at 1583 cm^−1^ as a function of retinoic acid concentration in the cytoplasm of the BEpC and A549 cells.

**Figure 6:**
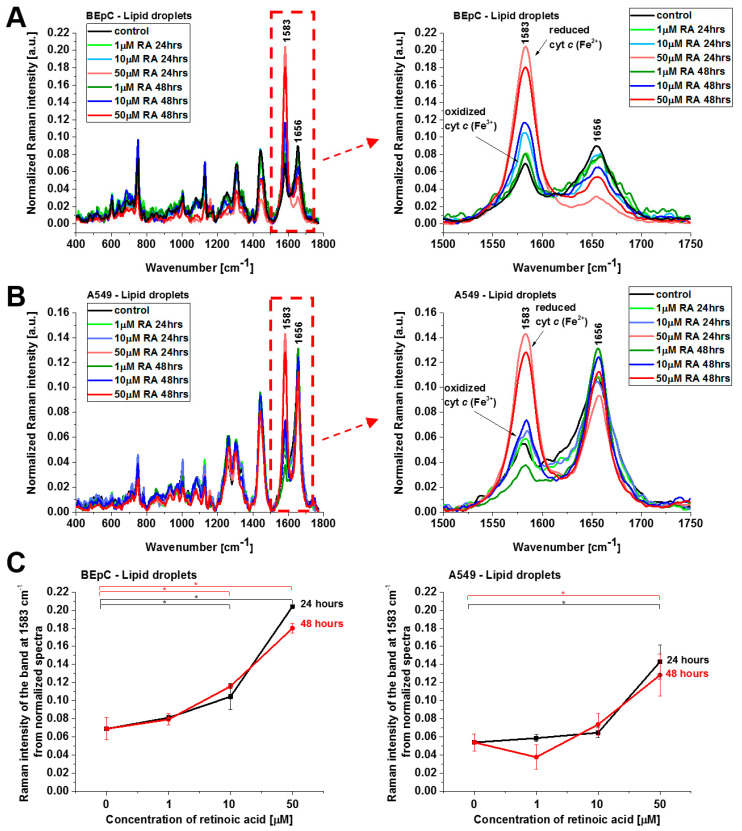
Normalized average Raman spectra of lipid droplets of human bronchial (BEpC) (**A**) and lung cancer cells (A549) (**B**) cells without and incubated with retinoic acid (1, 10, 50 μM) for 24 and 48 h. (**C**) Raman band ν_19_ intensity at 1583 cm^−1^ as a function of retinoic acid concentration in mitochondria of the BEpC and A549 cells.

The results presented so far demonstrate evidently that retinoic acid catalyzes a shift from the oxidized to the reduced form of cyt *c* in the mitochondrion. To further explore the mechanism governing the redox status of cyt *c* and the role of retinoic acid, we determined the redox status of cyt *c* in specific organelles. We measured the Raman spectra in the nucleus, endoplasmic reticulum (ER), lipid droplets, mitochondrion, cytoplasm, and membrane. Figure 5 and Figure 6 show normalized average Raman spectra (normalized by vector norm) for the cytoplasm and lipid droplets of a human bronchial (BEpC) and cancer lung cell (A549) without (control) and incubated with retinoic acid at a concentration of 1, 10, and 50 μM for 24 and 48 h.

Figure 5 shows the Raman spectra of cyt *c* in cytoplasm. Again, a dramatic increase in the Raman signal of the ν_19_ band at 1583 cm^−1^ of cyt *c* is observed upon incubation with retinoic acid. This enhancement illustrates the change in the redox status of the heme group from the oxidized iron ion Fe^3+^ to the reduced Fe^2+^.

Cyt *c*, released from mitochondria into the cytosol, triggers formation of the apoptosome, resulting in activation of caspases. This paper reviews the evidence for and against the redox state of cyt *c* regulating apoptosis, and possible mechanisms of this. Three research groups have found that the oxidized form of cyt *c* (Fe^3+^) can induce caspase activation via the apoptosome, while the reduced form (Fe^2+^) cannot [22]. The results from Figure 5 shows that cyt *c,* already in the reduced form, escapes from its natural location of mitochondria block apoptosis in cytosol when the cell detects a stimulus of retinoic acid.

**Figure 7:**
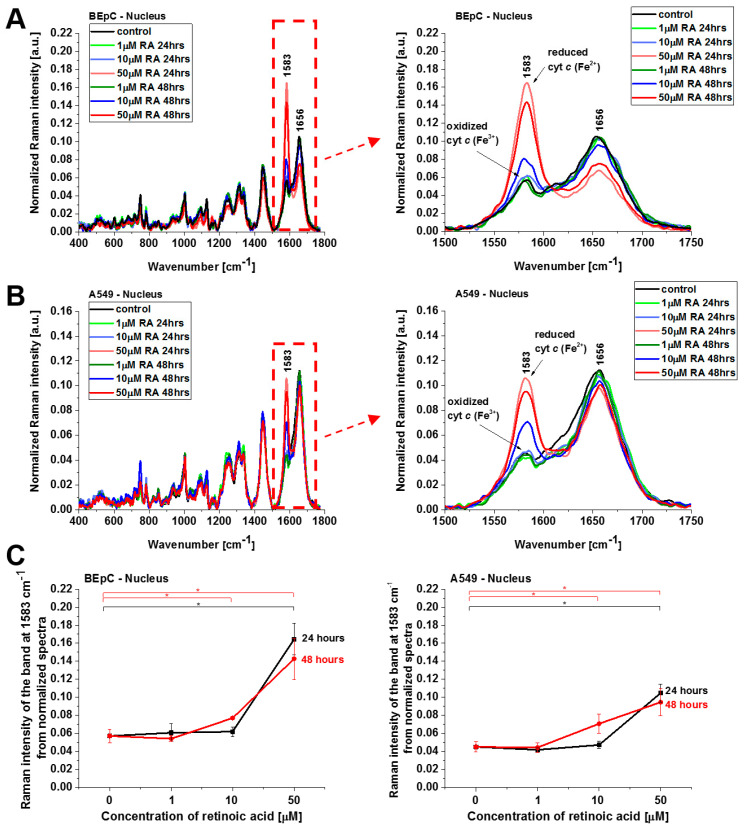
Normalized average Raman spectra of the nucleus of human bronchial (BEpC) (**A**) and lung cancer cells (A549) (**B**) cells without and incubated with retinoic acid (1, 10, 50 μM) for 24 and 48 h. (**C**) Raman band ν_19_ intensity at 1583 cm^−1^ as a function of retinoic acid concentration in the nucleus of the BEpC and A549 cells.

Cyt *c* in the reduced form is also observed in lipid droplets (Figure 6), ER, and nucleus (Figure 7). The concentration of the reduced form of cyt *c* is particularly high in lipid droplets and ER. The results for ER are particularly interesting in the view of the results presented by González-Arzola et al., where it was shown that early released cyt *c* binds to inositol 1,4,5-triphosphate receptors on the ER membrane, which stimulates further massive cyt *c* release from mitochondria [17].

Retinoic acid in cells is synthesized from vitamin A (retinol). Because humans are unable to produce vitamin A, they obtain retinol in a diet directly in food of animal origin or in the form of carotenoids from plants or retinyl esters derived from animal products [23,24]. 

Carotenoids and retinal esters are converted into retinol that is transported to other cells and tissues through lymphatic or blood circulation [25,26].

The transport of retinol (ROH) to the interior of the cells occurs via extracellular retinol acceptor—retinol-binding protein (RBP) that is present in the blood. Upon binding of RBP-ROH complex to the extracellular moiety of the transmembrane STRA6 protein (Stimulated by Retinoic Acid 6 protein or vitamin A receptor), retinol is transferred to apo-CRBP-1 (cellular retinol-binding protein 1) attached to the intracellular side of STRA6. The transfer of retinol from extracellular RBP-ROH to an intracellular acceptor apo-CRBP-1 triggers phosphorylation/activation of JAK2, a cytosolic non-receptor tyrosine kinases enzyme (non-receptor TK) that is bound to apo-CRBP-1 [27]. Inside the cytosol of the cell, retinol can be converted into retinyl esters in a reaction catalyzed by lecithin retinol acyltransferase (LRAT) to serve as cellular storage in lipid droplets, in a reaction catalyzed by LRAT [28].

**Figure 8:**
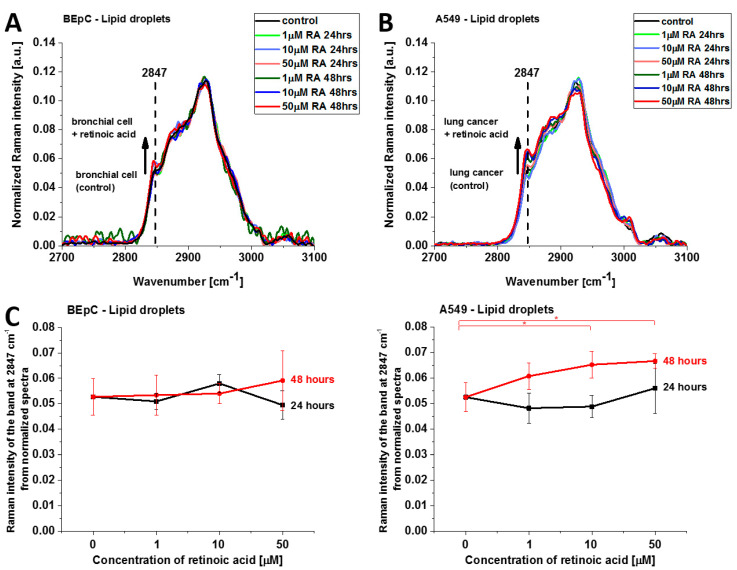
Normalized average Raman spectra of lipid droplets in human bronchial (BEpC) (**A**) and lung cancer cells (A549) (**B**) incubated with retinoic acid (1, 10, 50 µM) for 24 and 48 h and control (without retinoic acid). (**C**) Raman band intensity at 2847 cm^−1^ as a function of retinoic acid concentration in lipid droplets of the BEpC and A549 cells.

Figure 8 shows normalized average Raman spectra (2700–3100 cm^−1^) of lipid droplets in a typical BEpC and A549 incubated with retinoic acid (1, 10, 50 µM) for 24 and 48 h and control (without retinoic acid). The Raman band represents the mixed triglycerides-retinyl esters biochemical profile [6]. The peak at 2847 cm^−1^ represents vibrations typical for triglycerides. One can see that the Raman intensity that reflects the concentration of triglycerides increases upon incubation with retinoic acid. Detailed inspection into Figure 8A,B demonstrates that the concentration of triglycerides is higher in cancer lung cells than in normal lung cells because the Raman band at 2847 cm^−1^ is more intense for cancerous than normal cells. Recently, we found that triglycerides accumulate in lipid droplets for brain and breast cancers, whereas retinyl esters prevail in normal cells [6]. The results from Figure 8A,B support the previous conclusion and extend them to lung cancers.

This finding may indicate that retinoic acid induces a more aggressive phenotype of human lung cancer with enhanced lipid synthesis de novo [29].

**Figure 9:**
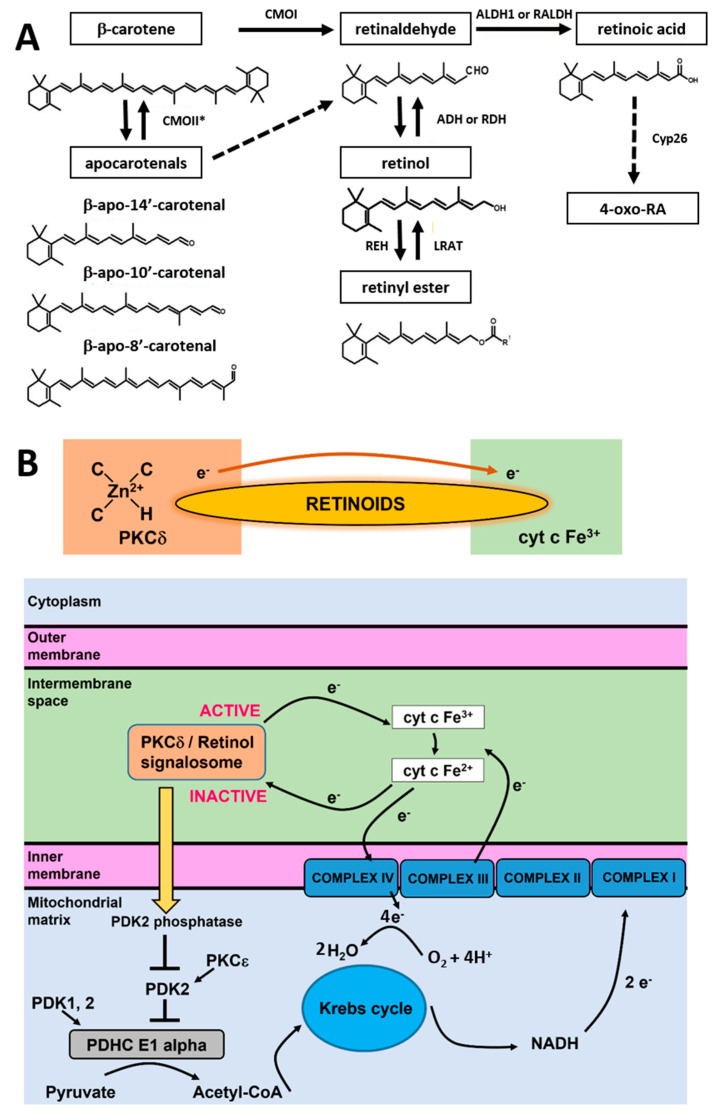
Summary of β-carotene, retinoids, and cytochromes metabolism [30,31] (**A**) and mechanism of redox regulation by the PKCδ/Retinol signalosome located in the mitochondrial intermembrane space based on Hammerling [5] (**B**).

In living cells, retinoic acid is generated in a two-step oxidative pathway and the concentration of retinoic acid in human tissue depends on a few families of enzymes presented in Figure 9A. First, β-carotene is transformed by CMOII enzyme into apocarotenals. The second channel is believed to be more important for cancers. β-carotene is cleaved by an enzyme of β-carotene 15,15′-oxygenase (CMOI) into two molecules of retinal (retinaldehyde) [30]. Retinal is catalyzed by the alcohol dehydrogenase (ADH) family to generate retinol, LRAT converts retinol into retinyl esters. Retinal catalyzed by RALDH enzyme forms RA (transcriptionally active), which can be metabolized by enzymes that belong to the cytochrome P450 (CYP) 26 family into more polar compounds, including 4-oxo retinoic acid, which is believed to be transcriptionally inactive [31]. The last step controls retinoic acid intracellular levels to maintain the appropriate concentration and consequent tissue distribution. Cytochrome P450 (CYP) subfamily 26 of enzymes degrade the excess of retinoic acid to avoid detrimental effects.

Once generated in the cell, retinoic acid begins to play its major function as a molecule essential for signaling. One of the signaling functions occurs in the nucleus where retinoic acid is transferred by a cellular retinoic acid-binding protein 2 (CRABP2) [32]. Upon entering the nucleus, retinoic acid (RA) interacts with specific nuclear transcription factors known as retinoic acid receptors (RARs), including three primary subtypes: RARα, RARβ, and RARγ [33]. Subsequently, RARs form heterodimers with Retinoid X receptors (RXRα, RXRβ, and RXRγ) [33], which recognize specific DNA sequences referred to as Retinoic acid response elements (RAREs) in the promoter region of RA target genes [34]. The RA-RAR-RXR complex binds to RAREs and induces conformational changes that facilitate the replacement of co-repressors with co-activators. Nuclear receptor co-activators (NCOA) recruit histone acetylases and trithorax proteins, resulting in chromatin relaxation and the activation of gene transcription [35]. Conversely, in the absence of RA, RAR-RXR heterodimers bind to RARE and recruit nuclear receptor co-repressors (NCOR). NCOR, in turn, recruits repressive factors like histone deacetylases and polycomb repressive complex 2, leading to chromatin condensation and gene silencing [35].

The RBP–retinol/STRA6/JAK2/STAT5/RA-RAR-RXR/STAT3/5 signaling cascade that regulates nuclear transcription is not able to explain the alterations in redox status of cyt *c* in the electron transport chain that are presented in the paper. The shift from the oxidized to the reduced form of cyt *c* has very serious consequences. First, the reduced cytochrome in mitochondria that is generated upon retinoic acid cannot activate the apoptosome or initiate apoptosis and cytokine induction once released to cytoplasm. In other words, it cannot play the role of a universal DAMP (damage-associated molecular patterns) able to alarm the immune system for danger in any type of cell or tissue by aborting the activation of the conventional mitochondrial signaling protein-dependent pathways. Moreover, the excess of reduced cyt *c* in the electron transport chain in mitochondria indicates the risk of overload in the electron transfer chain and mitochondrial dysfunctionality in electron flux flow between the complexes III and IV, enhanced ROS production, inhibition in the process of triggering ATP synthase by the electrostatic gradient in the mitochondrion inner membrane, and less efficient oxidative phosphorylation. The phosphorylation unit combines oxygen and hydrogen to produce H_2_O and ATP molecules. Hydrogen atoms are known as reducing equivalents. The passage of hydrogen atoms along the respiratory chain is equivalent to the passage of electrons through sequential redox reactions along protein complexes I-IV of the electron transfer chain, where O_2_ is reduced to H_2_O [36,37]. In detail, cytochrome c oxidase (complex IV) catalyzes the transfer of electrons from the reduced cyt *c* produced by complex II to molecular oxygen (O_2_). Its reduction to two water molecules requires four electron transfers from cyt *c* together with four protons, which are taken from the mitochondrial matrix (Figure 9B).

To explain the origin of retinoic acid effects and how they affect cyt *c* activities, we proposed the model based on the quantum chemistry models of retinoid biology based on PKCδ-retinoic acid complex catalysis resonance energy transfer (RET) reactions [5].

It has been proposed how retinol catalyzes RET reactions.^23^ The paper suggests that RET is pivotally important for mitochondrial energy homeostasis by controlling oxidative phosphorylation. The key role in this process is played by protein kinase C δ (PKCδ). PKCδ triggers a signal to the pyruvate dehydrogenase complex. The PKCδ-retinol complex reversibly responds to the redox potential of cyt *c*, which changes with the electron transfer chain flux. To understand the role of retinoids in cellular signal transduction, we combined our results presented in this paper with the mechanism of redox regulation by the PKCδ/Retinol signalosome presented in Figure 9B.

The model proposed by Hammering et al. [5] was derived originally for PKCδ/Retinol, but our results suggest the mechanism is even more effective for PKCδ/Retinoic acid. The PKCδ/Retinoids complex is located in the mitochondrial intermembrane space. The PKCδ signal complex consists of the protein kinase Cδ, the adapter protein Src homologous-collagen homolog (p66Shc), cyt *c*, and retinoic acid. The retinoid molecule binds PKCδ on a site in close proximity to its zinc-coordinated structure. It was suggested in ref. [5] that a conserved tryptophane residue enables resonance energy transfer [38] from PKCδ to the retinoid molecule. The retinoid molecule is believed to catalyze electron transfer to cyt *c* (Fe^3+^), initiating an unfolding process of PKCδ that yields the active form of the enzyme able to inactivate PDK2. The electron transfer from PKCδ via retinoid molecule to oxidized cyt *c* (Fe^3+^) makes cyt *c* reduced (Fe^2+^). Inactivation of PDK2 by the PKCδ/Retinoid molecule signalosome enhances the activity of pyruvate dehydrogenase phosphatases (PDK1,2) that effectively dephosphorylates, and thereby activates the E1 alpha regulatory domain of PDHC, leading to enhanced production of Acetyl-CoA from Pyruvate. Acetyl-CoA enters the Krebs cycle, driving the generation of NADH and increased electron transfer to complex III containing both cyt *c* as well as cyt *b*. The increased electron flow towards complex III gradually displaces the route associated with the signalosome-mediated reduction in oxidized cyt c, initiating a reversal of redox polarity. This reversal allows for the return of electrons to oxidized PKCδ. As the reduced and inactive form of PKCδ is reinstated, the signaling to PDHC weakens, resulting in a reduction in fuel flux. This reduction, in turn, mitigates the risk of overloading in the electron transfer chain.

This theoretical hypothesis is presented in ref. [5] received experimental support from our results presented in the paper.

In view of the results presented in the paper, one can state that retinoic acid induces enhanced production of reduced Fe^2+^ cyt *c*. The enhanced electron flux to cyt *c* from unbalanced PKCδ/Retinoid molecule signalosome spectacularly increases the reduced form of cyt c both in normal and in cancer cells. This effect is probed by the Raman intensity at 1583 cm^−1^ because the reduced cyt *c* (Fe^2+^) has a much higher intensity of the Raman bands than the oxidized cyt *c* (Fe^3+^) [8]. Consequently, the rise in Raman signal at 1583 cm^−1^ following incubation with retinoic acid indicates conversion from oxidized cyt *c* to reduced cyt *c.* We hypothesize that the process of redox regulation by the PKCδ/Retinoic acid signalosome is reversible only at normal physiological conditions. In contrast, during cancer development, the process of RET irreversibly activates PKCδ. Consequently, while capable of triggering the exergonic activating pathway, retinoids fail to activate the endergonic silencing path, trapping PKCδ in the ON position and causing harmful levels of ROS. The switch from reversible to irreversible RET mechanism in cancers is unclear, but we suggest that it may be related to the apocarotenoids, the primary products of the mitochondrial β-carotene, 9′-10′-oxygenase. It was reported by Hammerling et al. [5] that apocarotenoids have specific features, including irreversible modulation of energy homeostasis.

It is possible that competition between CMOI and CMOII enzymes (illustrated in Figure 9A) decide about reversibility or irreversibility of the RET mechanism in cancers.

## 4. Conclusions

We demonstrated that Raman imaging provides an excellent tool to extend our knowledge on the redox status of iron ion of heme proteins inside specific organelles of human bronchial and human lung cancer cells. We determined the redox status of cyt *c* regulated by retinoic acid in mitochondria, nucleus, lipid droplets, and cytoplasm in the bronchial normal and lung cancer cells. We showed that cell response to retinoic acid modulates mitochondrial metabolism towards its dysfunctions that favor the development of redox-dependent pathologies such as cancer. Here we report that the balance between oxidized and reduced cyt *c* is spectacularly shifted towards the reduced form in human lung cells upon incubation with retinoic acid. This shift results in modifications of cyt *c* functionality in oxidative phosphorylation, oxidative stress, and apoptosis.

The role of retinoic acid in regulating respiration, apoptosis processes, and ROS production has been discussed. We showed that retinoic acid plays a key role in the oscillations between Fe^2+^ and Fe^3+^ of cyt *c* in the electron transport chain that can activate or inhibit these physiological functions. We propose that a key modulator of mitochondrial activity is cyt *c* regulated by PKCδ-retinoic acid signal complex. Retinoic acid is instrumental in catalyzing resonance energy transfer reactions that play a pivotal role in maintaining mitochondrial energy balance through protein kinase C δ (PKCδ). PKCδ, in its function, communicates with the pyruvate dehydrogenase complex, regulating oxidative phosphorylation. Notably, the PKCδ-retinol complex can reversibly respond to shifts in the redox potential of cyt c, a parameter influenced by the workload of the electron transfer chain. The new role of retinoic acid in the change of the redox status of iron ion in the heme group of cyt from oxidized Fe^3+^ to reduced Fe^2+^ form has important consequences in reducing oxidative phosphorylation, aborting the activation of the conventional mitochondrial signaling protein-dependent pathways as well as lack of triggering programmed cell death through apoptosis. The redox status of cyt *c* regulated by retinoic acid becomes a target for further development of robust therapeutic approaches.

## Data Availability

The raw data underlying the results presented in the study are available from Lodz University of Technology Institutional Data Access. Request for access to those data should be addressed to the Head of the Laboratory of Laser Molecular Spectroscopy, Institute of Applied Radiation Chemistry, Lodz University of Technology. Data requests might be sent by email to the secretary of the Institute of Applied Radiation Chemistry: mitr@mitr.p.lodz.pl.

## Abbreviations

ADH, alcohol dehydrogenase; apo-CRBP-1, cellular retinol-binding protein 1; ATP, adenosine triphosphate; ATRA, all-trans retinoic acid; BEpC, human normal bronchial epithelial lung cell; CMOI, β-carotene 15,15′-oxygenase; CRABP2, cellular retinoic acid-binding protein 2; cyt *c*, cytochrome *c*; CYP, cytochrome P450; DAMP, damage-associated molecular patterns;ER, endoplasmic reticulum; ETC, electron transport chain; FADH2, flavin adenine dinucleotide; IFN1, type-1 interferon; IMS, intermembrane space of mitochondrion; LRAT, lecithin retinol acyltransferase; NADH, nicotinamide adenine dinucleotide; NCOA, nuclear receptor co-activators; NCOR, nuclear receptor co-repressors; p66Shc, Src homologous collagen homolog; PDHc, pyruvate dehydrogenase complex; PKCδ, protein kinase C δ; PRR, pattern recognition receptor; RA, retinoic acid; RARs, retinoic acid receptors; RARE, retinoic acid response element; RET, resonance energy transfer, RBP, retinol-binding protein; RIG-I, retinoic acid-inducible gene I; ROH, retinol; ROS, reactive oxygen species; STRA6 protein, stimulated by retinoic acid 6 protein or vitamin A receptor.
